# Circular RNA circKIF4A facilitates the malignant progression and suppresses ferroptosis by sponging miR-1231 and upregulating GPX4 in papillary thyroid cancer

**DOI:** 10.18632/aging.203172

**Published:** 2021-06-21

**Authors:** Wenkuan Chen, Jianchang Fu, Yingle Chen, Yudong Li, Li Ning, Dou Huang, Shumei Yan, Quan Zhang

**Affiliations:** 1 Department of Head and Neck, Sun Yat-Sen University Cancer Center, State Key Laboratory of Oncology in South China, Collaborative Innovation Center of Cancer Medicine, Guangzhou, China; 2 Department of Pathology, Sun Yat-Sen University Cancer Center, State Key Laboratory of Oncology in South China, Collaborative Innovation Center of Cancer Medicine, Guangzhou, China

**Keywords:** circKIF4A, circular RNAs, GPX4, competitive endogenous RNAs, papillary thyroid cancer

## Abstract

Circular RNAs (circRNAs) are one type of non-coding RNA. They act as important role in regulating various biological processes in the malignant progression. But we don’t clearly know the specific mechanism of the majority circRNAs in papillary thyroid tumor progression. In the current study, we explored circKIF4A and the result showed that it had high expression in papillary thyroid cancer. The functions of circKIF4A were explored by CCK-8, transwell, and mouse xenograft experiments. Knockdown of circKIF4A could suppress papillary thyroid cell growth and migration. In addition, RIP assays and dual luciferase vector reporter assays were further conducted. Our consequence showed circKIF4A facilitated the malignant progress of papillary thyroid tumor by sponging miR-1231 and upregulating GPX4 expression. In conclusion, our study proved that circKIF4A-miR-1231-GPX4 axis played a vital role in cancer proliferation and ferroptosis by competing endogenous RNAs. Therefore, targeting circKIF4A is very likely to be a potential method for treatment of papillary thyroid cancer in the future.

## INTRODUCTION

Based on the cancer statistics in the world, thyroid cancer is the fifth most common malignancy. It was predicted that there would be 52,070 cases of thyroid cancer in 2020, with 37,810 cases in female and 14,260 cases in male [1]. There are various subtypes in thyroid cancer, and 85%-90% of them are papillary thyroid cancer [2]. Approximately 90% of patients can be cured with standard treatment [3]. However, locoregional recurrences or distant metastases occur in almost 10% of thyroid carcinoma cases, which is still a challenge in the treatment of thyroid cancer [4]. Once recurrence and metastasis occur, the prognosis and the life quality of these patients will become worse. Consequently, it is extremely urgent to illustrate the specific mechanism of the carcinogenesis and invasion of papillary thyroid cancer.

Circular RNAs (circRNAs) are formed by covalently closed loops, which are regarded as novel endogenous noncoding RNAs [5]. They originate in the back-splice of pre-mRNAs (precursor mRNAs) without a head or a tail which are abundant in mammalian tissues [6]. Compared with linear counterparts, circRNAs show high stability *in vivo* because of their unique circular structure [7]. As important mediators of different biological process in the cell, circRNAs regulate the expression of vital genes via multiple comprehensive molecular mechanisms, including binding microRNAs (miRNAs), interacting with proteins and encoding novel polypeptides [8]. Plenty of circRNAs has been discovered as regulators of a diversity of diseases, including Alzheimer's disease, heart failure, diabetes, and cancers [9–13]. Masses of circRNAs has been characterized and validated to be mediators of the malignant progress with the development made in high-throughput circRNA sequencing [14, 15]. For instance, the most thoroughly studied circRNA ciRS-7 promotes the growth, invasion, drug resistance, and immune escape of various tumors by sponging miR-7 [16–21]. CircFBXW7 is low-expressed in tumor tissues. By translating a 21kDa novel protein (FBXW7-185aa) and sponging miRNA, circFBXW7 can suppress cell multiplication and invasion in glioma and breast cancer [22, 23]. Additionally, circKIF4A and circRAD18 can compete endogenous RNAs to regulate triple-negative breast cancer [24, 25]. CircHIPK3 promotes the development of colorectal cancer via upregulating a series of robust oncogenes (FAK, IGF1R, EGFR, YY1) [26]. Nevertheless, we still wonder whether circRNAs are crucial in papillary thyroid cancer.

In the current study, we explored how circKIF4A affected papillary thyroid cancer. We found circKIF4A was high-expressed in papillary thyroid tumor, while suppression of circKIF4A led to the low growth and migration. Generally, we confirmed that the circKIF4A-miR-1231-GPX4 axis was associated with the malignant progress of papillary thyroid cancer.

## RESULTS

### circKIF4A is upregulated in papillary thyroid cancer with circular characteristics

**Figure 1:**
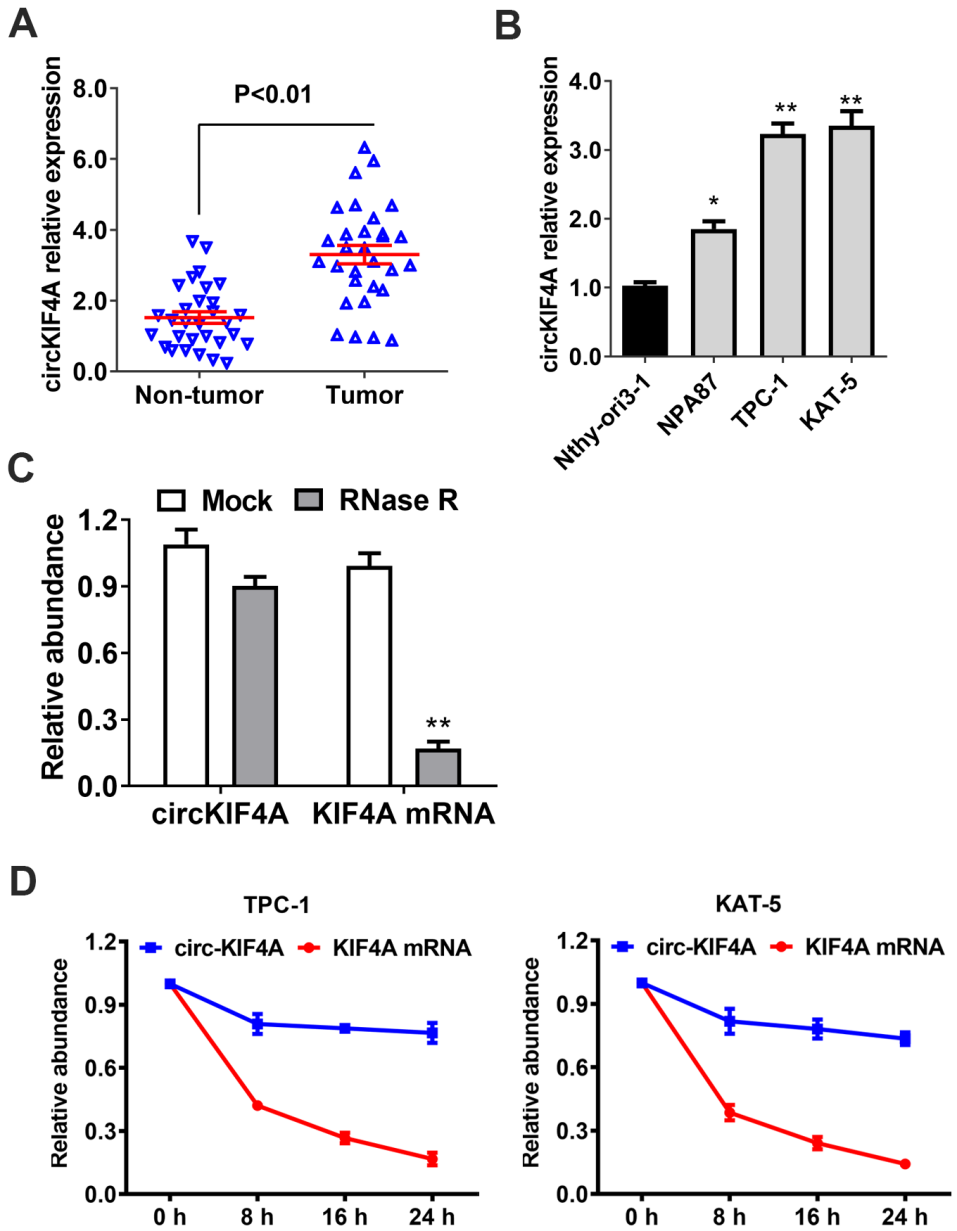
**circKIF4A is upregulated in papillary thyroid cancer with circular characteristics.** (**A**) circKIF4A expression in adjacent normal tissues and papillary thyroid cancer. (**B**) The relative expression of circKIF4A in cell lines. (**C**) RNase R assay examined the circular structure of circKIF4A in TPC-1 cell line. (**D**) Circular transcripts of KIF4A (circKIF4A) was more stable than its linear mRNA transcripts determined by actinomycin D treated assay.

First, we used RT-qPCR to analyze whether circKIF4A has different expression in 30 pairs of papillary thyroid cancer tissues and the nearby normal thyroid tissues. circKIF4A was found upregulated in papillary thyroid cancer (Figure 1A). Besides, compared to Nthy-ori3-1 (normal thyroid cell), especially in TPC-1 and KAT-5 cell lines, we found circKIF4A was upregulated (Figure 1B). Then, the circular structure and stability of circKIF4A was further examined by specific assays. In RNase R digestion experiment, circKIF4A was resisted to RNA exonuclease while linear KIF4A mRNA was digested after incubated with RNase R in TPC-1 cell (Figure 1C). In consistent, in actinomycin D assays, we found the circular form of circKIF4A had longer half-life span than the linear KIF4A mRNA (Figure 1D).

### Knockout of circKIF4A attenuates the proliferation of papillary thyroid cancer cells

**Figure 2:**
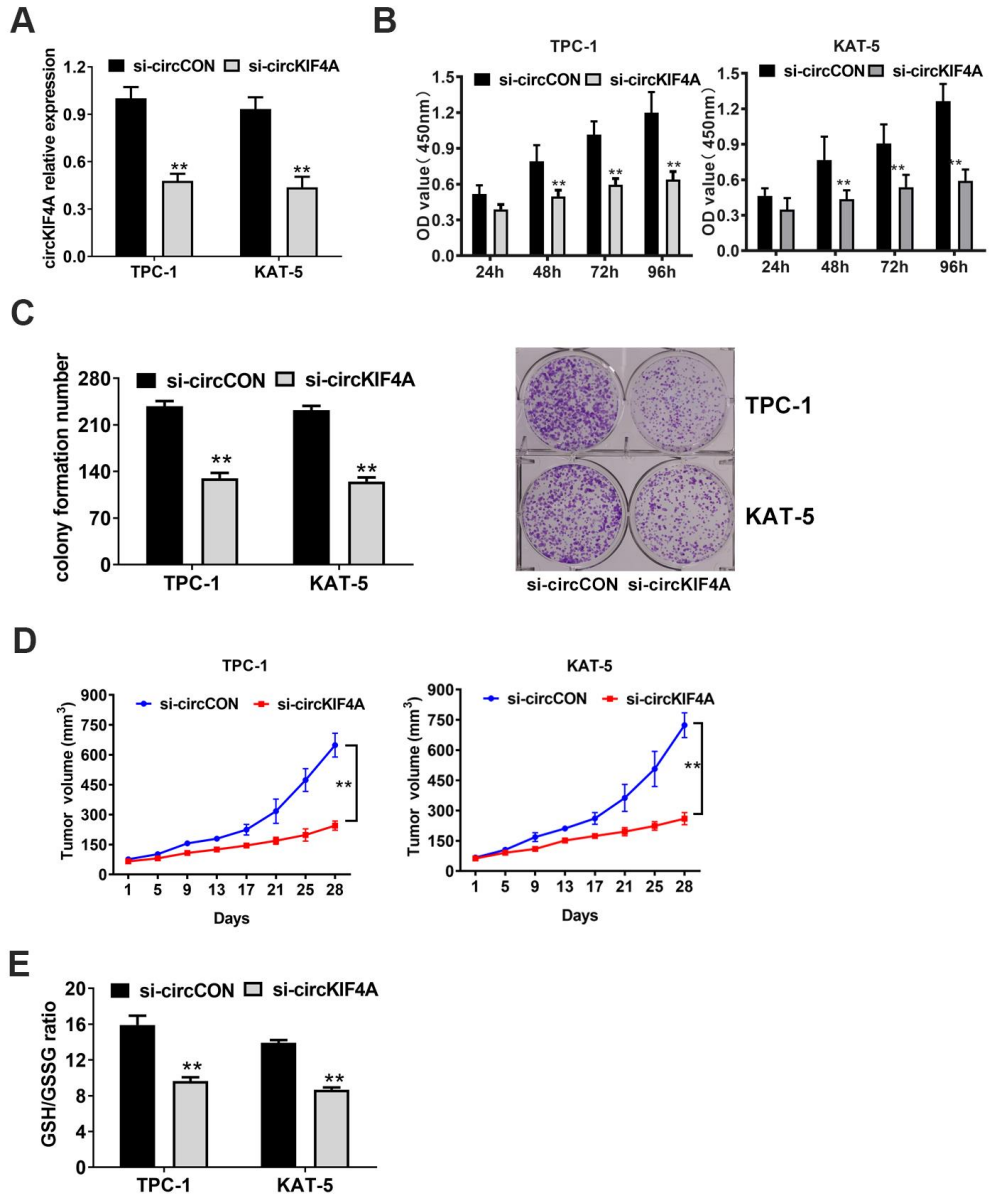
**Knockdown of circKIF4A attenuates the proliferation of papillary thyroid cancer cells.** (**A**) Knockdown effect of circKIF4A was assessed in TPC-1 and KAT-5 cell line. (**B**) CCK-8 assays evaluated cell proliferation after knockdown of circKIF4A. (**C**) Colony formation assays revealed that circKIF4A silencing suppressed cell colony formatting ability. (**D**) Mouse xenograft models were established. Tumor volume was estimated in every four days. (**E**) GSH/GSSG ratio was detected.

We used loss-of-function assays to figure out the function of circKIF4A. An siRNA was designed to silence circKIF4A by targeting the back-splicing junction region of circRNA. Validating by RT-qPCR analysis, circKIF4A had decreased after siRNA transfection, which showed the efficacy of the knockdown assay (Figure 2A). We found downregulation of circKIF4A attenuated proliferation in CCK-8 assays (Figure 2B). Similarly, circKIF4A silencing suppressed cell colony formatting ability, according to the colony formation assays (Figure 2C). Besides, we established mouse xenograft models to explore whether circKIF4A plays a role *in vivo*. We measured the tumor volumes at each time point, and the result showed that depletion of circKIF4A could inhibit tumor growth (Figure 2D). Reduction of the glutathione (GSH)/oxidized glutathione (GSSG) ratio was observed after knockdown of circKIF4A (Figure 2E).

### Downregulation of circKIF4A inhibits the metastasis of papillary thyroid cancer cells

**Figure 3:**
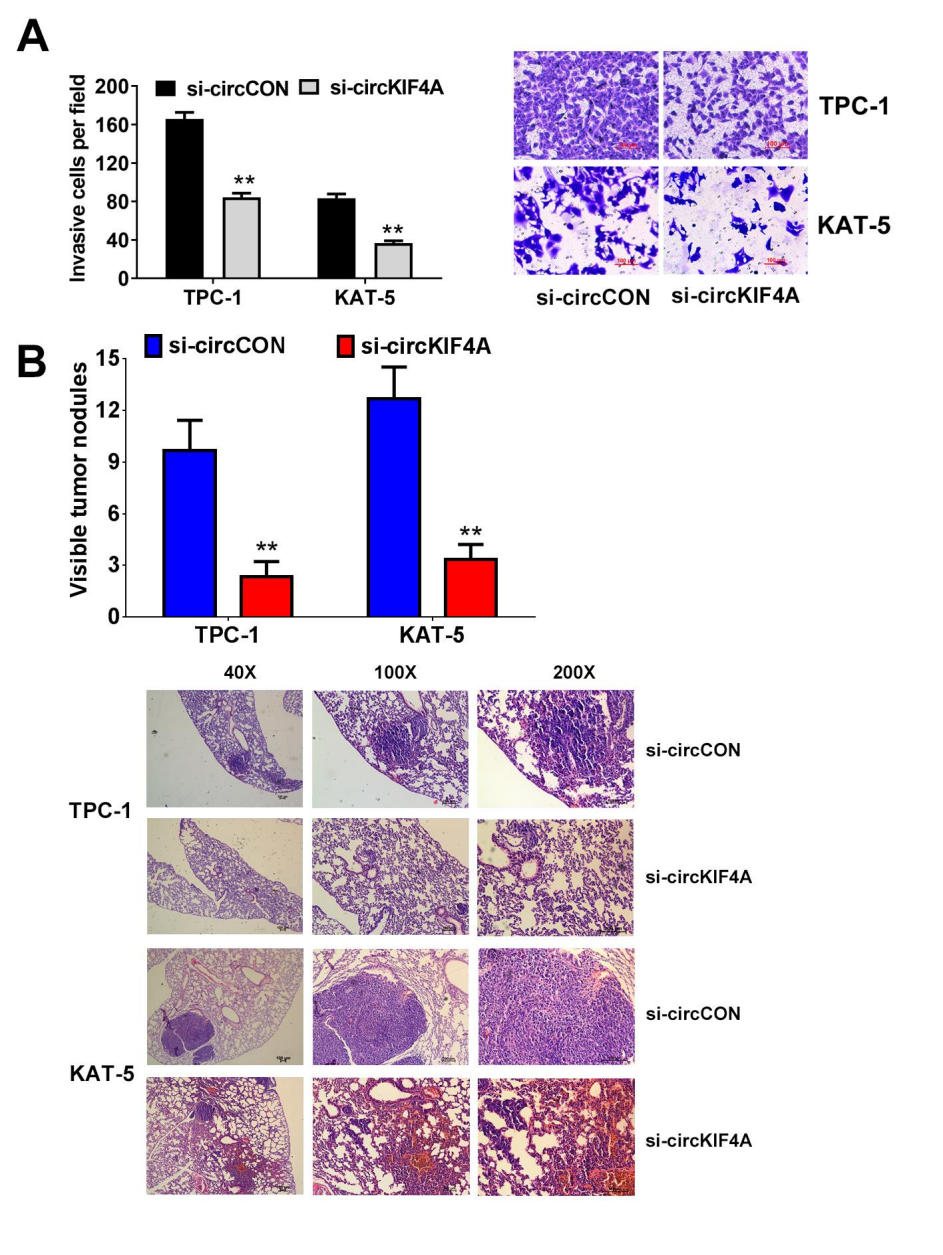
**Downregulation of circKIF4A suppresses the metastasis of papillary thyroid cancer cells.** (**A**) Transwell experiments were conducted in TPC-1 and KAT-5 cell line. (**B**) The number of lung metastases was counted and recorded. HE-stained sections of lung metastases were presented.

We next performed assays to investigate if the metastasis of papillary thyroid cancer is influenced by circKIF4A. The result showed that cells had low activity to migrate by silencing the expression of circKIF4A (Figure 3A). In consistent with this result, suppression of circKIF4A could also inhibit the metastasis in lung metastasis experiment *in vivo*, indicating that circKIF4A is vital in the metastasis of thyroid cancer (Figure 3B).

### circKIF4A acts as a sponge of miR-1231 in papillary thyroid cancer

**Figure 4:**
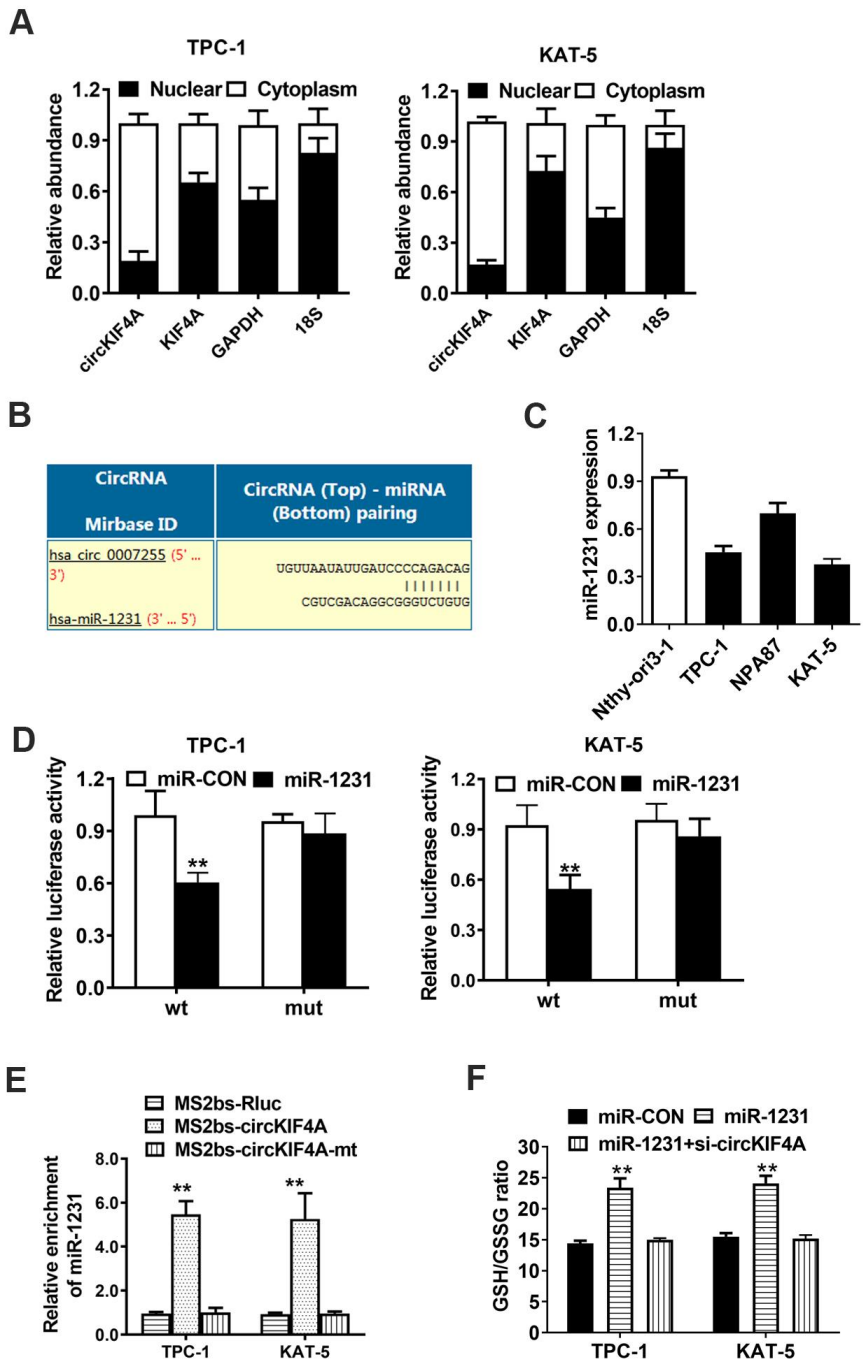
**circKIF4A acts as a sponge of miR-1231 in papillary thyroid cancer.** (**A**) 18S, GAPDH, circKIF4A and KIF4A in nuclear and cytoplasmic part analyzed by RT-qPCR. (**B**) Predicted interaction site of miR-1231 within the circKIF4A sequence. (**C**) miR-1231 expression in papillary thyroid cancer cell lines. (**D**) Luciferase reporter assay of TPC-1 and KAT-5 cells transfected with miR-1231 mimics and circKIF4A. (**E**) MS2-based RIP assay transfected with MS2bs-circKIF4A, MS2bs-circKIF4A-mt or Rluc control. (**F**) GSH/GSSG ratio was detected. GSH/GSSG ratio was increased after overexpression of miR-1231 which could be reversed by silencing circKIF4A.

Next, we determined the subcellular location of circKIF4A by isolating of the cytoplasmic and nuclear portions of cellular RNA. The result showed that circKIF4A predominantly existed in the cytoplasm, which might indicate that it interacted with miRNA in the cytoplasm (Figure 4A). We furtherly used Circular RNA Interactome to predict whether there is a potential interaction between circRNA and miRNAs. Among the candidates, we found miR-1231 might have the ability to bind circKIF4A sequence (Figure 4B). By RT-qPCR analysis, we found there was a decreasing tendency of miR-1231 in papillary thyroid cancer cell lines (Figure 4C). The transfection of WT reporter and miR-1231 caused the deactivation of relative luciferase, according to dual luciferase reporter assays (Figure 4D). Then, we conducted RIP assays to prove the direct relationship between circKIF4A and miR-1231. We also noticed that miR-1231 was mainly enriched in the MS2bs-circKIF4A group (Figure 4E). Overexpression of miR-1231 could increase the GSH level which could be reversed by silencing circKIF4A in TPC-1 and KAT-5 papillary thyroid cancer cells (Figure 4F).

### CircKIF4A promotes papillary thyroid cancer progression through circKIF4A-miR-1231-GPX4 axis

**Figure 5:**
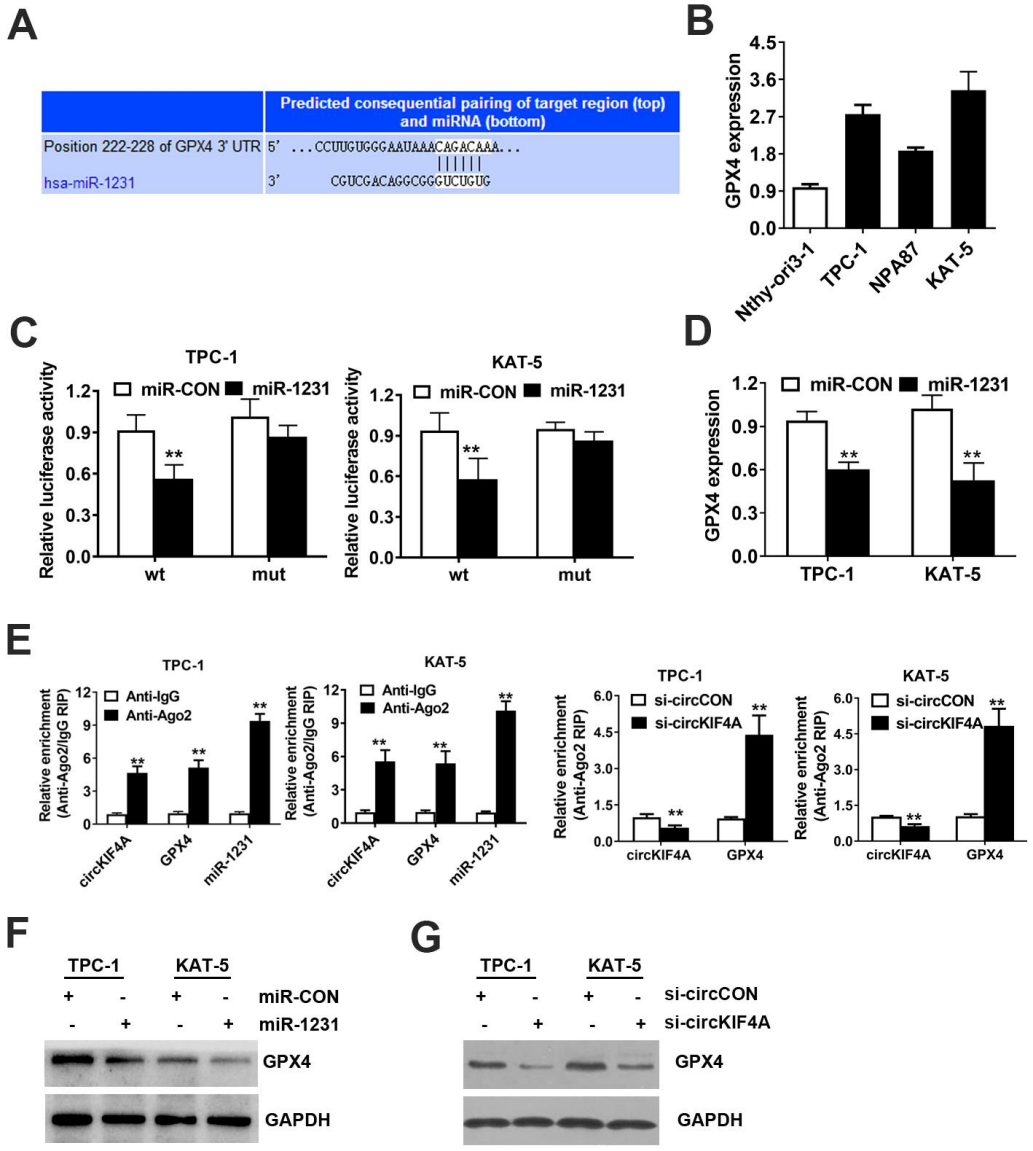
**CircKIF4A promotes papillary thyroid cancer progression through circKIF4A-miR-1231-GPX4 axis.** (**A**) Predicted interacting site of miR-1231 within the 3’-UTR of GPX4. (**B**) GPX4 expression in papillary thyroid cell lines. (**C**) Dual luciferase reporter assay of TPC-1 and KAT-5 cells. (**D**) Expression of GPX4 was reduced after overexpression of miR-1231. (**E**) Enrichment of circKIF4A, GPX4 and miR-1231 on AGO2 RNA binding protein assessed by RIP assay. (**F**) Overexpression of miR-1231 resulted in the reduction of GPX4 protein expression. (**G**) Knockdown of circKIF4A reduced the protein expression of GPX4.

We used TargetScan to further find out the potential targets of miR-1231. We found GPX4 was a downstream target oncogene among the candidates (Figure 5A). GPX4 encodes a protein and protects cells against oxidative damage which plays an important role in multiple cancers, certainly including papillary thyroid cancer [27–31]. By RT-qPCR analysis, we found GPX4 overexpressed in papillary thyroid cancer cell lines, detected (Figure 5B). The next step was to explore whether miR-1231 could bind the 3’-UTR of GPX4 mRNA directly. We found that the relative luciferase activity of cells was significantly decreased after transfecting miR-1231 and WT 3’-UTR-GPX4 vector. However, this phenomenon was not observed when mutant vector was transfected (Figure 5C).

Additionally, miR-1231 mimics significantly reduced the expression level of GPX4 mRNA, which indicates that GPX4 is downregulated by miR-1231 (Figure 5D). Moreover, AGO2 related RIP assays were carried out. We found that circKIF4A, GPX4 and miR-1231 gathered to AGO2 RNA binding protein (Figure 5E). Suppression of circKIF4A could significantly increase GPX4 enrichment to RNA induced silencing complex (Figure 5E). Knockdown of circKIF4A could reduce the protein expression of GPX4, while inhibition of miR-1231 could lead to the reversion of the protein expression of GPX4, analyzed by western blot assay (Figure 5F). Knockdown of circKIF4A significantly decreased the expression level of GPX4 detected by western-blot analysis (Figure 5G).

## DISCUSSION

CircRNAs have become popular topics in RNA biological field and have attracted the eyes of many scientists. The high-throughput sequencing technology and bioinformatics analysis enabled scientists to discover and investigate various kinds of circRNAs [14, 15]. Researchers have come to realized that these special non-coding RNAs with circular structures are integral parts in multiple biological processes rather than worthless product of pre-mRNA splicing [32]. circPRKCI is upregulated in lung adenocarcinoma which promotes tumorigenesis by sponging miR-589 and miR-545 [33]. By translating a novel 370-aa β-catenin isoform, a circRNA derived from CTNNB1 pre-mRNA promotes the proliferation hepatocellular carcinoma cell with the mechanism of activating the Wnt signaling pathway [34]. circPLK1 were identified as oncogenic drivers by reducing apoptosis in breast cancer [35]. However, there are only few studies investigating the roles and functions of circRNAs in papillary thyroid cancer. For example, circ_0006156 had higher expression in serum exosomes, and through the miR-1178/TLR4 axis, it could accelerate papillary thyroid cancer to bad prognosis [36]. Through the FGFR1 pathway, circRAPGEF5 modulates progression of thyroid cancer [37].

In this study, the result showed circKIF4A was a frequently upregulated novel circRNA. Besides, knockout of circKIF4A could significantly inhibit the development of cancer *in vitro* and vivo. All the results showed that circKIF4A was able to directly sponge miR-1231 and promote papillary thyroid cancer progression by upregulating antioxidant protein GPX4 expression.

According to the reported studies, miR-1231 had suppressive effect in many cancers. For instance, miR-1231 is secreted by stem cells, it can prevent pancreatic cancer cells from growing and metastasizing [38]. By targeting EGFR expression, miR-1231 is downregulated in prostate cancer [39]. Additionally, miR-1231 can also lower the risk of papillary thyroid cancer cell [40]. As a target downstream of miR-1231, GPX4 protects cells against oxidative damage which is harmful to multiple cancers, certainly including papillary thyroid cancer. The GSH (glutathione)-GPX4 (glutathione peroxidase 4) system is considered to be a main cell protection system that suppresses ferroptosis [41, 42]. We confirmed GPX4 was the target of miR-1231 in papillary thyroid cancer. By competing endogenous RNA, circKIF4A could enhance the expression of GPX4.

In summary, we confirmed that the circKIF4A-miR-1231-GPX4 axis was associated with papillary thyroid cancer. It could compete endogenous RNA and suppress the progression and metastasis. Therefore, we might consider targeting circKIF4A as a novel method for treatment of papillary thyroid cancer.

## MATERIALS AND METHODS

### Clinical sample data and ethical standards

We collected fresh nearby normal thyroid tissues and primary papillary thyroid cancer samples from Sun Yat-Sen University Cancer Center. This study was approved by the Ethics Committee of the Sun Yat-Sen University Cancer Center. We collected all the written informed consent before doing this study.

### Cell culture

The cell lines used in this study were all purchased from the ATCC. Cell lines were cultured according to the supplier’s instructions. Cell authenticity was verified occasionally by the method of DNA fingerprinting.

### CCK-8 assay

3000 si-circKIF4A and si-circCON cells were put into a 96-well plate. The cells were incubated for each time period in the incubator at 37° C. Afterwards, CCK-8 solution (10ul) was added and incubate for one hour before measurement. Basic information is listed in (Supplementary Table 1).

### Western blot analysis

RIPA lysis and PMSF were used to isolate protein from cells. The protein was put into each well of the SDS-PAGE gel and separated. The protein was afterwards moved to the PVDF membranes for 2 hours at 300 mA. The membrane was treated with each antibody at 4° C overnight and then incubated with the specific secondary antibody at room temperature for 1 hour. Anti-GPX4 (1:1000, Abcam, USA) and anti-GAPDH antibody (1:1000, Affinity, USA) are used to detect certain protein.

### RNase R digestion assay

After 3 ug extracted total RNA of TPC-1 papillary thyroid cancer cells were treated with RNase R (5 Uug) or control solution for 20 min at 37° C, the resulting RNA solution was purified and quantified by RT-qPCR analysis.

### Actinomycin D assay

TPC-1 and KAT-5 cells were exposed with 2ugml actinomycin D to degrade the linear mRNA transcription for 0, 8, 16, and 24 hours. The TPC-1 and KAT-5 cells were harvested at certain time period and the linear KIF4A mRNA and circular circKIF4A were quantified by qPCR-analysis.

### RT-qPCR analysis

Total RNA was extracted by TRIzol (Invitrogen). qRT-PCR assays were carried out using Takara SYBR PCR kit. Basic information is listed in (Supplementary Tables 2, 3).

### Transwell assay

Totally, 5×10^4^ cells were resuspended and added to the above chambers (serum-free medium) and medium (medium with 20% FBS) was loaded to the lower chambers. Methanol was utilized to fix the left cells. The migrated cells were imaged and counted, after staining with crystal violet (1%).

### Luciferase reporter assay

TPC-1 and KAT-5 cells were seeded 1 × 10^4^ cells in each well in 96-well plates. circKIF4A and GPX4 3’-UTR was cloned into a CMV promoter-driven luciferase in a pCDNA3.0 vector after amplified from human genomic DNA. The putative miRNA binding site of circKIF4A and GPX4 3’-UTR was mutated in luciferase reporter assays. The constructed reporting vectors (circKIF4A-wt/mut or GPX4 3’-UTR-wt/mut) and miRNA inhibitors or mimics were both transfected into cells for 48 hours.

### RNA immunoprecipitation (RIP)

Cells were transfected with MS2bs-circKIF4A, MS2bs-circKIF4A-mt and MS2bs-Rluc. After incubating for 48 hours, RIP was performed. The relative level of miR-1231 was determined after purification. The RIP assays for AGO2 were performed with an IP level anti-Ago2 antibody (Millipore). The abundance of circKIF4A, GPX4 mRNA and miR-1231 was tested after RNA purification.

### Mouse xenograft model

KAT-5 and TPC-1 cells (2×10^7^) were subcutaneously injected into nude mice (four mice/group, 4-week-old) and treated with intratumoral injection (50 μL si-circCON, si-circKIF4A) every four days. The volume of tumors was estimated every four days according to the formula 0.5×width^2^×length. After 28 days, the tumors were weighed. For *in vivo* lung metastasis assay, cells (1 × 10^5^) were injected through tail veins (four mice/group). The lungs were extracted after 8 weeks and the number of metastatic sites were quantified via microscopy of HE-stained sections.

### Statistical analysis

All statistical analysis was performed with SPSS 22.0 software. All data are reported as the mean ± standard deviation (SD). Groups were compared using Student’s t test. Paired t test was used to compare the expression of circKIF4A in two groups. *P*<0.05 was regarded as statistically significant.

## Supplementary Material

Supplementary Tables

## References

[1] Siegel RL, Miller KD, Jemal A. Cancer statistics, 2019. CA Cancer J Clin. 2019; 69:7–34. 10.3322/caac.21551 30620402

[2] Santiago K, Chen Wongworawat Y, Khan S. Differential MicroRNA-signatures in thyroid cancer subtypes. J Oncol. 2020; 2020:2052396. 10.1155/2020/2052396 32565797 PMC7290866

[3] Park H, Park J, Park SY, Kim TH, Kim SW, Chung JH. Clinical course from diagnosis to death in patients with well-differentiated thyroid cancer. Cancers (Basel). 2020; 12:2323. 10.3390/cancers12082323 32824662 PMC7463440

[4] Pelizzo MR, Merante Boschin I, Toniato A, Pagetta C, Casal Ide E, Mian C, Rubello D. Diagnosis, treatment, prognostic factors and long-term outcome in papillary thyroid carcinoma. Minerva Endocrinol. 2008; 33:359–79. 18923371

[5] Zhang XO, Wang HB, Zhang Y, Lu X, Chen LL, Yang L. Complementary sequence-mediated exon circularization. Cell. 2014; 159:134–47. 10.1016/j.cell.2014.09.001 25242744

[6] Jeck WR, Sorrentino JA, Wang K, Slevin MK, Burd CE, Liu J, Marzluff WF, Sharpless NE. Circular RNAs are abundant, conserved, and associated with ALU repeats. RNA. 2013; 19:141–57. 10.1261/rna.035667.112 23249747 PMC3543092

[7] Bach DH, Lee SK, Sood AK. Circular RNAs in cancer. Mol Ther Nucleic Acids. 2019; 16:118–29. 10.1016/j.omtn.2019.02.005 30861414 PMC6411617

[8] Wang Y, Hou J, He D, Sun M, Zhang P, Yu Y, Chen Y. The emerging function and mechanism of ceRNAs in cancer. Trends Genet. 2016; 32:211–24. 10.1016/j.tig.2016.02.001 26922301 PMC4805481

[9] Hansen TB, Kjems J, Damgaard CK. Circular RNA and miR-7 in cancer. Cancer Res. 2013; 73:5609–12. 10.1158/0008-5472.CAN-13-1568 24014594

[10] Shan K, Liu C, Liu BH, Chen X, Dong R, Liu X, Zhang YY, Liu B, Zhang SJ, Wang JJ, Zhang SH, Wu JH, Zhao C, Yan B. Circular noncoding RNA HIPK3 mediates retinal vascular dysfunction in diabetes mellitus. Circulation. 2017; 136:1629–42. 10.1161/CIRCULATIONAHA.117.029004 28860123

[11] Devaux Y, Creemers EE, Boon RA, Werfel S, Thum T, Engelhardt S, Dimmeler S, Squire I, and Cardiolinc Network. Circular RNAs in heart failure. Eur J Heart Fail. 2017; 19:701–09. 10.1002/ejhf.801 28345158

[12] Huang JL, Qin MC, Zhou Y, Xu ZH, Yang SM, Zhang F, Zhong J, Liang MK, Chen B, Zhang WY, Wu DP, Zhong ZG. Comprehensive analysis of differentially expressed profiles of Alzheimer’s disease associated circular RNAs in an Alzheimer’s disease mouse model. Aging (Albany NY). 2018; 10:253–65. 10.18632/aging.101387 29448241 PMC5842852

[13] Yu T, Wang Y, Fan Y, Fang N, Wang T, Xu T, Shu Y. CircRNAs in cancer metabolism: a review. J Hematol Oncol. 2019; 12:90. 10.1186/s13045-019-0776-8 31484561 PMC6727394

[14] Li S, Teng S, Xu J, Su G, Zhang Y, Zhao J, Zhang S, Wang H, Qin W, Lu ZJ, Guo Y, Zhu Q, Wang D. Microarray is an efficient tool for circRNA profiling. Brief Bioinform. 2019; 20:1420–33. 10.1093/bib/bby006 29415187

[15] Vo JN, Cieslik M, Zhang Y, Shukla S, Xiao L, Zhang Y, Wu YM, Dhanasekaran SM, Engelke CG, Cao X, Robinson DR, Nesvizhskii AI, Chinnaiyan AM. The landscape of circular RNA in cancer. Cell. 2019; 176:869–81.e13. 10.1016/j.cell.2018.12.021 30735636 PMC6601354

[16] Yang W, Gu J, Wang X, Wang Y, Feng M, Zhou D, Guo J, Zhou M. Inhibition of circular RNA CDR1as increases chemosensitivity of 5-FU-resistant BC cells through up-regulating miR-7. J Cell Mol Med. 2019; 23:3166–77. 10.1111/jcmm.14171 30884120 PMC6484300

[17] Yang W, Yang X, Wang X, Gu J, Zhou D, Wang Y, Yin B, Guo J, Zhou M. Silencing CDR1as enhances the sensitivity of breast cancer cells to drug resistance by acting as a miR-7 sponge to down-regulate REGγ. J Cell Mol Med. 2019; 23:4921–32. 10.1111/jcmm.14305 31245927 PMC6652952

[18] Memczak S, Jens M, Elefsinioti A, Torti F, Krueger J, Rybak A, Maier L, Mackowiak SD, Gregersen LH, Munschauer M, Loewer A, Ziebold U, Landthaler M, et al. Circular RNAs are a large class of animal RNAs with regulatory potency. Nature. 2013; 495:333–38. 10.1038/nature11928 23446348

[19] Weng W, Wei Q, Toden S, Yoshida K, Nagasaka T, Fujiwara T, Cai S, Qin H, Ma Y, Goel A. Circular RNA ciRS-7-A promising prognostic biomarker and a potential therapeutic target in colorectal cancer. Clin Cancer Res. 2017; 23:3918–28. 10.1158/1078-0432.CCR-16-2541 28174233 PMC5511556

[20] Zou Y, Zheng S, Deng X, Yang A, Kong Y, Kohansal M, Hu X, Xie X. Diagnostic and prognostic value of circular RNA CDR1as/ciRS-7 for solid tumours: a systematic review and meta-analysis. J Cell Mol Med. 2020; 24:9507–17. 10.1111/jcmm.15619 32783378 PMC7520288

[21] Zou Y, Zheng S, Deng X, Yang A, Xie X, Tang H, Xie X. The role of circular RNA CDR1as/ciRS-7 in regulating tumor microenvironment: a pan-cancer analysis. Biomolecules. 2019; 9:429. 10.3390/biom9090429 31480381 PMC6770779

[22] Yang Y, Gao X, Zhang M, Yan S, Sun C, Xiao F, Huang N, Yang X, Zhao K, Zhou H, Huang S, Xie B, Zhang N. Novel role of FBXW7 circular RNA in repressing glioma tumorigenesis. J Natl Cancer Inst. 2018; 110:304–15. 10.1093/jnci/djx166 28903484 PMC6019044

[23] Ye F, Gao G, Zou Y, Zheng S, Zhang L, Ou X, Xie X, Tang H. circFBXW7 inhibits Malignant progression by sponging miR-197-3p and encoding a 185-aa protein in triple-negative breast cancer. Mol Ther Nucleic Acids. 2019; 18:88–98. 10.1016/j.omtn.2019.07.023 31536884 PMC6796723

[24] Tang H, Huang X, Wang J, Yang L, Kong Y, Gao G, Zhang L, Chen ZS, Xie X. circKIF4A acts as a prognostic factor and mediator to regulate the progression of triple-negative breast cancer. Mol Cancer. 2019; 18:23. 10.1186/s12943-019-0946-x 30744636 PMC6369546

[25] Zou Y, Zheng S, Xiao W, Xie X, Yang A, Gao G, Xiong Z, Xue Z, Tang H, Xie X. circRAD18 sponges miR-208a/3164 to promote triple-negative breast cancer progression through regulating IGF1 and FGF2 expression. Carcinogenesis. 2019; 40:1469–79. 10.1093/carcin/bgz071 31001629

[26] Zeng K, Chen X, Xu M, Liu X, Hu X, Xu T, Sun H, Pan Y, He B, Wang S. CircHIPK3 promotes colorectal cancer growth and metastasis by sponging miR-7. Cell Death Dis. 2018; 9:417. 10.1038/s41419-018-0454-8 29549306 PMC5856798

[27] Peng G, Tang Z, Xiang Y, Chen W. Glutathione peroxidase 4 maintains a stemness phenotype, oxidative homeostasis and regulates biological processes in Panc-1 cancer stem-like cells. Oncol Rep. 2019; 41:1264–74. 10.3892/or.2018.6905 30535490

[28] Liu Y, Wang Y, Liu J, Kang R, Tang D. Interplay between MTOR and GPX4 signaling modulates autophagy-dependent ferroptotic cancer cell death. Cancer Gene Ther. 2021; 28:55–63. 10.1038/s41417-020-0182-y 32457486

[29] Heirman I, Ginneberge D, Brigelius-Flohé R, Hendrickx N, Agostinis P, Brouckaert P, Rottiers P, Grooten J. Blocking tumor cell eicosanoid synthesis by GP x 4 impedes tumor growth and malignancy. Free Radic Biol Med. 2006; 40:285–94. 10.1016/j.freeradbiomed.2005.08.033 16413410

[30] Ubellacker JM, Tasdogan A, Ramesh V, Shen B, Mitchell EC, Martin-Sandoval MS, Gu Z, McCormick ML, Durham AB, Spitz DR, Zhao Z, Mathews TP, Morrison SJ. Lymph protects metastasizing melanoma cells from ferroptosis. Nature. 2020; 585:113–18. 10.1038/s41586-020-2623-z 32814895 PMC7484468

[31] Viswanathan VS, Ryan MJ, Dhruv HD, Gill S, Eichhoff OM, Seashore-Ludlow B, Kaffenberger SD, Eaton JK, Shimada K, Aguirre AJ, Viswanathan SR, Chattopadhyay S, Tamayo P, et al. Dependency of a therapy-resistant state of cancer cells on a lipid peroxidase pathway. Nature. 2017; 547:453–57. 10.1038/nature23007 28678785 PMC5667900

[32] Chen LL. The biogenesis and emerging roles of circular RNAs. Nat Rev Mol Cell Biol. 2016; 17:205–11. 10.1038/nrm.2015.32 26908011

[33] Qiu M, Xia W, Chen R, Wang S, Xu Y, Ma Z, Xu W, Zhang E, Wang J, Fang T, Hu J, Dong G, Yin R, et al. The circular RNA circPRKCI promotes tumor growth in lung adenocarcinoma. Cancer Res. 2018; 78:2839–51. 10.1158/0008-5472.CAN-17-2808 29588350

[34] Liang WC, Wong CW, Liang PP, Shi M, Cao Y, Rao ST, Tsui SK, Waye MM, Zhang Q, Fu WM, Zhang JF. Translation of the circular RNA circβ-catenin promotes liver cancer cell growth through activation of the Wnt pathway. Genome Biol. 2019; 20:84. 10.1186/s13059-019-1685-4 31027518 PMC6486691

[35] Kong Y, Yang L, Wei W, Lyu N, Zou Y, Gao G, Ou X, Xie X, Tang H. CircPLK1 sponges miR-296-5p to facilitate triple-negative breast cancer progression. Epigenomics. 2019; 11:1163–76. 10.2217/epi-2019-0093 31337246

[36] Wu G, Zhou W, Pan X, Sun Z, Sun Y, Xu H, Shi P, Li J, Gao L, Tian X. Circular RNA profiling reveals exosomal circ_0006156 as a novel biomarker in papillary thyroid cancer. Mol Ther Nucleic Acids. 2020; 19:1134–44. 10.1016/j.omtn.2019.12.025 32059339 PMC7016027

[37] Liu W, Zhao J, Jin M, Zhou M. circRAPGEF5 contributes to papillary thyroid proliferation and metastatis by regulation miR-198/FGFR1. Mol Ther Nucleic Acids. 2019; 14:609–16. 10.1016/j.omtn.2019.01.003 30785065 PMC6379567

[38] Shang S, Wang J, Chen S, Tian R, Zeng H, Wang L, Xia M, Zhu H, Zuo C. Exosomal miRNA-1231 derived from bone marrow mesenchymal stem cells inhibits the activity of pancreatic cancer. Cancer Med. 2019; 8:7728–40. 10.1002/cam4.2633 31642612 PMC6912060

[39] Wang Y, Zhang Q, Guo B, Feng J, Zhao D. miR-1231 is downregulated in prostate cancer with prognostic and functional implications. Oncol Res Treat. 2020; 43:78–86. 10.1159/000504606 31822000

[40] Pan Y, Xu T, Liu Y, Li W, Zhang W. Upregulated circular RNA circ_0025033 promotes papillary thyroid cancer cell proliferation and invasion via sponging miR-1231 and miR-1304. Biochem Biophys Res Commun. 2019; 510:334–38. 10.1016/j.bbrc.2019.01.108 30709584

[41] Yang WS, SriRamaratnam R, Welsch ME, Shimada K, Skouta R, Viswanathan VS, Cheah JH, Clemons PA, Shamji AF, Clish CB, Brown LM, Girotti AW, Cornish VW, et al. Regulation of ferroptotic cancer cell death by GPX4. Cell. 2014; 156:317–31. 10.1016/j.cell.2013.12.010 24439385 PMC4076414

[42] Xu T, Ding W, Ji X, Ao X, Liu Y, Yu W, Wang J. Molecular mechanisms of ferroptosis and its role in cancer therapy. J Cell Mol Med. 2019; 23:4900–12. 10.1111/jcmm.14511 31232522 PMC6653007

