# Supplementary material for: Circular RNA circKIF4A facilitates the malignant progression and suppresses ferroptosis by sponging miR-1231 and upregulating GPX4 in papillary thyroid cancer
Source: Aging (Albany NY). 2021 Jun 21;13(12):16500–12. doi: 10.18632/aging.203172 (PMC8266339; doi:10.18632/aging.203172)
Supplement: Supplementary Tables [file aging-13-203172-s001.pdf]

SUPPLEMENTARY TABLES

Supplementary Table 1. The target sequences of siRNAs used in this study.

siRNA	Sequence (5' - 3')
si-circCON	UUCUCCGAACGUGUCACGUTT
si-circKIF4A	GAUCUAUAACGUAUUAAUATT

Supplementary Table 2. Primer sequences for qRT-PCRs used in this study.

Target	Direction	Sequence (5' - 3')
18S	Forward	TTAATTCCGATAACGAACGAGA
	Reverse	CGCTGAGCCAGTCAGTGTAG
circKIF4A	Forward	GAGGTACCCTGCCTGGATCT
	Reverse	TGGAATCTCTGTAGGGCACA
GPX4	Forward	GAGGCAAGACCGAAGTAAACTAC
	Reverse	CCGAACTGGTTACACGGGAA
miR-1231	Forward	CCTCAACTGAATTGCCGACTC
	Reverse	CTCAACTGGTGTCGTGGAGTC
GAPDH	Forward	GGAGCGAGATCCCTCCAAAAT
	Reverse	GGCTGTTGTCATACTTCTCATGG

Supplementary Table 3. The sequences of oligonucleotides used in this study.

Oligonucleotides	Sequence (5' - 3')
miR-1231 mimics	GUGUCUGGGCGGACAGCUGC
Control mimics	UCUACUCUUUCUAGGAGGUUGUGA
